# Reappraising the exteriorization of the mammalian testes through evolutionary physiology

**DOI:** 10.1080/19420889.2019.1586047

**Published:** 2019-03-20

**Authors:** William B. Miller, Jr, John S. Torday

**Affiliations:** a Banner Health, Department of Medicine, Phoenix, AZ, USA; b Department of Pediatrics, Harbor-UCLA Medical Center, Torrance, CA, USA

**Keywords:** endothermy, balanced ecological hypothesis, exteriorization of the testes, scrotum, gubernaculum

## Abstract

A number of theories have been proposed to explain the exteriorization of the testicles in most mammalian species. None of these provide a consistent account for the wide variety of testicular locations found across the animal kingdom. It is proposed that testicular location is the result of coordinate action of testicular tissue ecologies to sustain preferential states of homeostatic equipoise throughout evolutionary development in response to the advent of endothermy.

## Introduction

The origin of exteriorization of the testes is unknown. Nor is there any complete agreement about its phylogeny. Some have suggested that the scrotum evolved prior to the evolution of mammals, and then was intermittently lost [1]. Others have insisted that the scrotum evolved at least twice, having been lost by some species and then regained [2]. There has been a general assumption that the emergence of endothermy accounts for that differential testicular location but there are many disagreements as to exact causes among various clades and species [2–11]. It is proposed that a reconstruction of the critical pathways towards endothermy can clarify the wide variety of testicular locations in animals. In particular, as only mammals and birds are endothermic, there is an opportunity to compare their developmental and phylogenic evolution with that of other organisms based on molecular-cellular and cell–cell interactions [12]. Comparative analysis of their response to endothermy permits a coherent explanation of their respective testicular locations and the variable position of the testes in other animals. The critical factor in this reappraisal is the recognition that testicular tissue ecologies can be active participants in their own evolutionary development. From this non-traditional stance, it is further argued that a similar ecological perspective can offer insight into the etiology of infertility and elevated rates of testicular cancer in crytorchidism.

## Theories of the origin of the exteriorization of the testes

In 1926, Moore asserted that the evolution of the scrotum was an evolutionary adaptation to provide an appropriate cool environment of spermatogenesis in response to endothermia [13]. In theory, higher body temperatures hamper sperm production and increase rates of spontaneous mutation. Therefore, a scrotal location would improve sperm production and would also lower the rate of spontaneous mutations. This is of particular importance since the rate of spontaneous mutation rates is higher in the male germ line than the female, particularly affecting the Y chromosome [14,15]. Freeman (1990) proposed the ‘training hypothesis’ as a different evolutionary accommodation [3]. Testicular descent is a mechanism for improved sperm quality through the imposition of relative hypoxia within the scrotal sac, causing stress-induced enhanced oxidative metabolism that could result in increased gametic aerobic fitness. Bedford (1978) and Jones (2002) indicated otherwise [16,17]. They suggested an alternative ‘cool storage hypothesis’. The cooling requirements of the epididymis were the key issue in testicular descent in support of sperm storage and as a site of gametic optimization in preparation for sperm competition. While these particulars remain theoretical, it is clear is that the epididymis is critical for mammalian sperm maturation. It is a highly specific microenvironment for the movement of ions, organic solutes, proteins, and a wide variety of critical hormones such as androgens, estrogens, and retinoids that are necessary for reproduction [8].

In apposition to prior theories, Portman (1952) suggested that a scrotum and exterior testes were an opportunity for sexual display to attract females, accounting for its ornamentation among some species [18]. This led to the ‘handicap hypothesis’ which postulated the substantive purpose of costly sexual ornamentation was as a representative signal of overall fitness to members of the opposite sex [9]. Only members of a group that were highly fit could survive the relative handicap of prominent sexual ornamentation. However, any attempts to provide experimental evidence of the condition-dependent expression of sexual ornamentation in support of the handicap hypothesis have proved disappointing [6].

In order to try to make sense of the variety of scrotal positions in differing animal groups, Chance (1996) offered the ‘galloping hypothesis’. Animals whose mobility is characterized by quick movements or jumping, such as horses, primates, and humans have external testes to avoid concussive hydrostatic rises in intra-abdominal pressure [4]. Elephants, whose testicles are internal, do not jump. According to that theory, the testes adjusted to cooler exterior scrotal temperatures as a secondary adaptation.

Two other theories have gained particular purchase. In the ‘activation hypothesis’, descended scrotal testicles might have evolved as a situation-specific mechanism for activating sperm through consistent differences in temperature between the male and female reproductive tracts [9]. A 2–3 centigrade temperature difference links to the activation of sperm in the higher body temperature of the vagina that accompanies insemination. This theory also attempts to explain the cremasteric reflex as a coital reaction drawing the testicle closer to the abdomen and its higher temperature during sexual arousal to improved sperm motility in preparation for ejaculation. The main problem with this theory is that many mammals do not have descended testicles. For example, elephant testicles remain close to their originating peri-renal location throughout life. In recognition of this disconnection, the ‘endothermic pulse hypothesis’ attempted an explanation [11]. It was argued that the evolution of the scrotum was driven by increases in physiological body temperature (endothermic pulses) that occurred in Boreoeutheria, a clade of diverse mammals that includes rodents, primates, rabbits, bats, and ungulates. The model proposes that fitness favored scrotal testes by maintaining an optimum temperature for spermatogenesis and sperm storage that mirrored the successful adaptation that had occurred throughout the Cenozoic at the lower levels of body temperature that prevailed in ancestral mammals for at least 163 million years. Cooler testicular temperatures for spermatogenesis are favored biologically as the optimum condition for spermatogenesis, sperm storage, and a lowered rate of mutation. The hypothesis does offer an explanation for why elephants do not have external testes. Elephants are not part of Boreoeutheria but instead, belong to a different clade, Afrotheria, which also includes moles, shrew, aardvarks and manatees. Yet, to complicate matters, marsupials seem to have evolved their scrotum entirely independently from placental mammals [2]. In part, this surmise rests on the observation that the testes of marsupials are in front of their penises [19].

Despite all diversity of opinions, one bedrock assumption undergirds each of them. Testicular location is a product of selection pressures. The major difficulty within that presumption is the exact means of accounting for testes location through selection bias when the conditions for mature testicular location are so varied among species. For example, birds do not have external testes. They maintain a very high body temperature, up to 108 degrees, which on the basis of theory should disfavor spermatogenesis [20,21]. Elephants with abdominal testes have a higher core body temperature than gorillas or marsupials that have external testes [22,23]. Although the evolution of testicular position is believed to have been affected by selection pressures to protect male gonads from physical trauma, there is no reason to suppose that one set of animals is more affected than another [2]. Although most mammals have descended scrotal testes, others do not. Some have ‘descended ascrotal’ testes in which the testes emerges from the abdominal wall, but do not move into a scrotum, instead, staying just under the skin, a condition seen in whales, pinnipeds (seals and walruses), sloths, moles, and rhinoceros [24]. Other animals demonstrate ‘testicondy’ in which the testes do not move out of the abdominal cavity and stay close to the kidneys. This group includes the Afrotheria clade of elephants, manatees, and the duck-billed platypus and other monotremes. In theory, all should have low core body temperatures for spermatogenesis, but only monotremes do [24].

Therefore, no consensus exists as to the origin of scrotal testes that is entirely coherent, or, for that matter, for the location of other parts of the testicular apparatus since the location of the critical epididymis also seems to be highly idiosyncratic on an evolutionary basis. Some animals with testicondy extend their epididymis to a sub-cutaneous location presumably providing a cooling effect during sperm storage and maturation [16]. In such circumstances, testes location would have to be regarded as secondary to epididymal requisites.

## The origin of endothermy

### Effect of duplication of the βadrenergic receptor

Since it is proposed that the cellular-molecular adjustment to endothermy is crucial to the testicular position of animals, a thorough detailed **e**xploration of a causal mechanism for the evolution of endothermy through cellular-molecular mechanisms and novel cell-cell signaling pathways is warranted. Those cellular accommodations were propelled by novel genes in the hypothalamic-pituitary-adrenal axes (HPA) of both mammals and birds in response to contemporaneous changes in atmospheric oxygen levels and specific key gene duplications during the water–land transition [25–29]. Although both birds and mammals achieved endothermy, significant physiological and anatomic differences emerged, including testicular location. There has been a general assumption that the emergence of endothermy accounts for that differential testicular location but there are many disagreements as to exact causes among various clades and species [2–11].

During the course of lung evolution in adaptation to land, based on the step-wise cellular-molecular remodeling of the lung alveoli, there were opportunities to reduce the diameter of the alveoli in order to increase the surface area-to-blood volume ratio, increasing gas exchange across the alveolar wall based on the Law of Laplace [30,31]. The underlying cause of such remodeling was due to increased gravitational force on land increasing blood pressure, resulting in increased shear stress on the microvasculature [32]. This generated Radical Oxygen Species known to cause gene mutations and duplications [33]. Such remodeling would then have had a direct influence on the levels of homeostasis that constrain processes of cellular variation [34].

In tandem with the structural change in alveolar diameter, the βAdrenergic Receptor duplicated due to the excessive stress on the evolving lung, episodic hypoxia being the most potent physiologic agonist for the Hypothalamic-Pituitary-Adrenal (HPA) Axis [26,28]. The duplication/amplification of the βAdrenergic Receptor during the transition from water to land facilitated local control of lung blood pressure, rendering it independent of systemic blood pressure control. This was a requirement for adaptation to land; the advent of independent βAdrenergic regulation of the pulmonary and systemic blood pressures relieved the evolutionary constraint on the alveolar microvasculature to support that adaptation [35].

**Figure 1.**
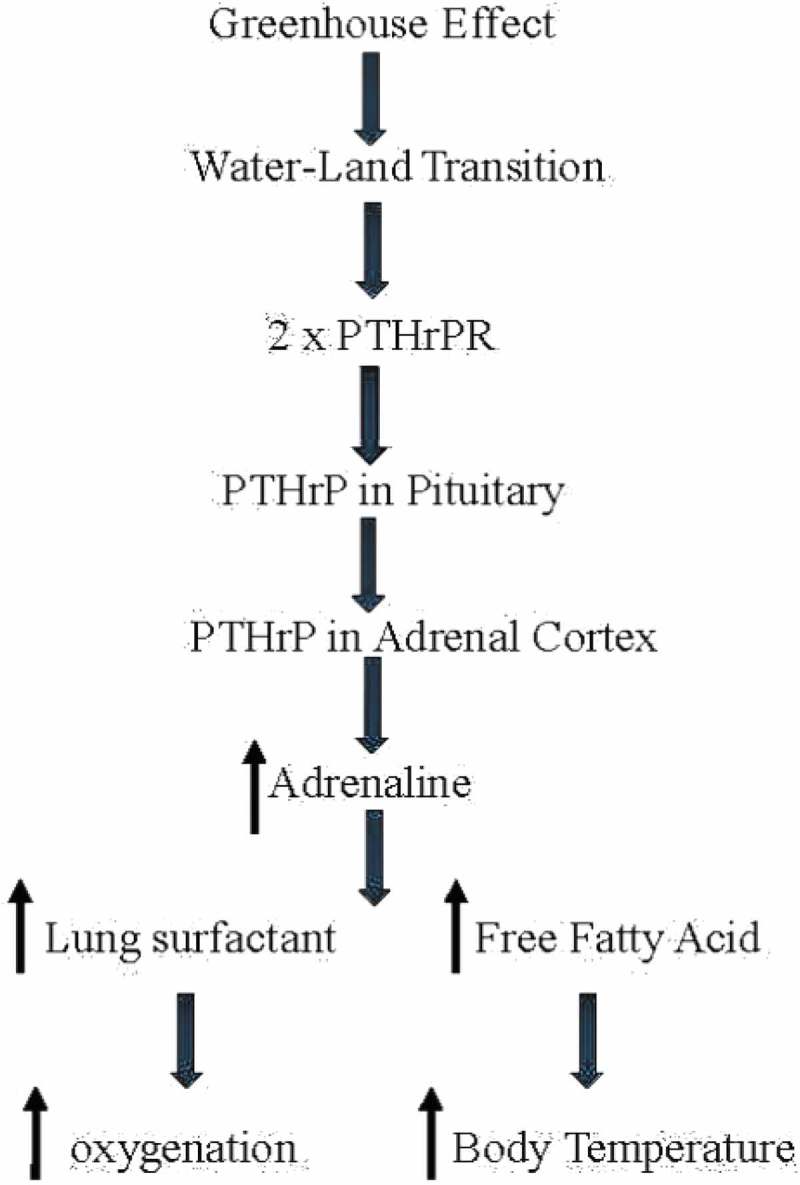
**The evolution of endothermy**. Romer’s ‘Greenhouse Effect’ dried up bodies of water, forcing vertebrates on to land. During that period, the parathyroid Hormone-related Protein Receptor (PTHrPR) duplicated. PTHrP signaling appeared in the anterior pituitary, enhancing ACTH production; PTHrP signaling also appeared in the adrenal cortex, enhancing corticoid production. That stress-induce cascade increased adrenaline production, increasing production of lung surfactant in the alveoli, alleviating the constraint of hypoxia; it also increased the constraint of hypoxia; it also increased the production of free fatty acids from fat pads, increasing body temperature.

Based on the Romer Hypothesis, land vertebrates emerged from water about 400 million years ago in response to the ‘greenhouse’ effect of rising levels of carbon dioxide in the atmosphere, drying up bodies of water globally (Figure 1).

Based on the fossil record, vertebrates breached land on at least five separate occasions, indicating the magnitude and direction of the selection pressure to ‘Gain Ground’ [7,36,37]. Remarkably, no attention has been paid to the obligatory, concomitant evolution of the visceral organs involved during this key evolutionary transitional period, other than to document the phylogenetic differences between fish, amphibians, reptiles, mammals, and birds. The disconnect between such phenotypic observations and the underlying mechanisms of evolution is due to the unequivocal emphasis placed on random mutation and natural selection by conventional Darwinian evolutionists. In contrast to this dogmatic approach, Torday and Rehan (2012) have pointed out the value added in determining the cellular–molecular adaptation to oxygenation in forming the mammalian lung through the specific cell–cell interactions that determine its embryonic development [34]. Such cellular mechanisms are mediated by soluble growth factors and their cognate receptors, acting in a reiterative manner to engineer cell-based solutions to environmental pressures, thereby generating form and function based on homeostatic principles [34]. It has been previously defended that such cellular–molecular interrelationships can be traced back to the crucial eukaryotic unicellular state by following the pathways generated by lipids in accommodating calcium homeostasis, and their consequent effects on oxygen uptake by cells, tissues, and organs [38,39].

### Atmospheric oxygen, physiologic stress, gene duplication, and lung evolution

A primary argument is that visceral organ changes are cellular adaptations to physiologic stress caused by the external environment that yields countervailing internal cellular accommodations. It is further asserted that these are consistently enacted at the level of the cellular ecologies that comprise any organ system. Based on the adaptive changes cited above, the consequences of cyclic fluctuations in environmental oxygen can be considered [7]. Initially, there was protection against environmental oxygen by sterol hopanoids intrinsic to prokaryotic bacteria [40]. Mechanistically, oxygen stimulates the SREBP/Scap family of enzymes that regulate sterol biosynthesis in prokaryotes and eukaryotes alike, reflecting the depth of this evolved trait. Konrad Bloch had hypothesized that the synthesis of cholesterol was due to the increased availability of atmospheric oxygen, since it takes eleven atoms of oxygen to make one molecule of cholesterol, however, bacteria do not produce cholesterol, so the oxygen-sterol connection must have had some other origin [41].

For example, Deamer (2017) has written extensively about the role of polycyclic hydrocarbons, thought to be omnipresent throughout the universe and foundational to the origins of life on Earth [42]. It is hypothesized that aromatic molecules delivered to the young earth during the heavy bombardment phase in the early history of our solar system were likely to be among the most abundant and stable organic compounds available. The *Aromatic World Hypothesis* suggests that aromatic molecules might function as energy transduction elements, templating genetic components for early life forms [43]. These molecules can experimentally stabilize fatty acid vesicles much like cholesterol does in contemporary cell membranes, and can foster the biosynthesis of nucleotides [44].

During the Phanerozoic period, much larger fluctuations in atmospheric oxygen, ranging between 12% and 35% are now widely recognized to have caused dramatic increases in animal body size [7,45]. What has not been addressed previously are the physiologic consequences of the concomitant, episodic decreases in oxygen that followed the increases documented by Berner [7].The effect of hypoxia, the most potent known physiologic stressor, is mediated by the HPA Axis in vertebrates. Pituitary ACTH stimulates corticoid production by the adrenal cortex which then promotes catecholamine production by the downstream adrenal medulla [46]. This physiologic mechanism is of evolutionary significance because catecholamines cause surfactant secretion from the lung alveoli. That increased production would have acutely alleviated the hypoxic stress on the lung by further reducing surface tension, increasing the distention of the alveolar wall. In turn, distension of the alveolar epithelium would have stimulated alveolar type II cell PTHrP production, coordinately increasing both the degree of alveolarization and alveolar vascular perfusion, since it is an angiogenic factor thus promoting comprehensive and coordinate physiologic increases in gas exchange surface area over the course of evolutionary time [36,47–49].

### Duplication of glucocorticoid receptor genes

In further support of this mechanism for physiologic adaptation, other gene duplications are known to have occurred during the water–land transition beside the βAdrenergic Receptor (βAR) and the Glucocorticoid Receptor (GR) [28,29]. Both were required to facilitate vertebrate land adaptation. The increase in βARs alleviated the constraint on pulmonary blood pressure independent of systemic blood pressure [35]. The GR evolved from the Mineralocorticoid Receptor (MR), likely due to the constraint of the orthostatic increase in blood pressure due to the increased force of gravity on land-adapting vertebrates. This was exacerbated by the effect of stress on mineralocorticoid stimulation of blood pressure, now offset by diverting some MR expression to the GR. Combined with the synergistic effect of adrenocortical glucocorticoid production on adrenomedullary βAR production, physiology was integrated for successful land adaptation [50].

It can be defended that increased PTHrP signaling in soft tissues such as the lung during the water–land transition would have favored those members of the species whose adaptation through higher levels of PTHrP facilitated bone adaptation [4]. Moreover, physiologic stress is known to cause microvascular capillary shear stress, which causes genetic mutations, including gene duplications [33]. It can be supposed that such effects, particularly on the nascent pulmonary microvasculature, would have been critical for land adaptation since increased respiration causes stress on the lung microvasculature.

### Evolution of endothermy/homeothermy as evidence for the effect of stress on vertebrate physiologic evolution

One can easily argue whether these physiologic adaptations were causal since there is no fossilized evidence for this sequence of events *per se*. Yet, it can be defended that the functional relationships that have been described are internally consistent insofar as their role is directed towards the steady maintenance of cellular homeostasis. There is also an *a priori* scenario for the subsequent evolution of these integrated physiologic traits that can be presented as internally consistent with the advent of endothermy/homeothermy. The above-mentioned gene duplications entail the exploitation of pre-existing physiologic traits that would permit the rise of endothermy/homeothermy to meet the environmental stresses of the water–land transition along antecedent pathways from which constructive physiological linkages could arise [25]. In the scenario cited above, the advantage of amplified catecholamine activity to alleviate the constraint of air-breathing would have secondarily caused the lipolytic secretion of fatty acids from peripheral fat cells [51]. In consequence, increasing metabolism and body temperature would be expected [52,53]. In turn, leptin secretion has been shown to increase the basal metabolic rate of some ectotherms, such as Fence Lizards, consistent with the putative role of adrenaline in the evolution of endothermy [54].

It is known that mammalian lung surfactant, composed of saturated phosphatidylcholine, functions at a level which is 300% more active in reducing surface tension at 37°C than at 25°C. This effect is due to the elevated phase transition temperature of saturated phosphatidylcholine (41°C) as the temperature at which a lung surfactant film would deteriorate and is no longer able to reduce surface tension. It is asserted that this co-evolution of saturated phosphatidylcholine production by the alveoli and endothermy/homeothermy may have been due to the pleiotropic effects of catecholamines, stimulating both surfactant secretion by the alveoli, and coordinately increasing the unsaturated fatty acid composition of peripheral cell membranes [25]. In consequence, there would be an increase in oxygen uptake through improved membrane fluidity. It can be argued that the progressive phylogenetic increase in the percentage of saturated phosphatidylcholine in lung surfactant is indicative of the constitutive change in adaptation to endothermy/homeothermy [55]. These fundamental changes in lipid composition in service to metabolism are exaptations of the events that initiated eukaryotic evolution [42]. Considering the severe conditions generated by Romer’s Gap, during which vertebrates were virtually wiped off the face of the Earth, it should not be surprising that such deep homologies were recruited during this critical phase of vertebrate evolution [56].

It has recently been hypothesized that among amniotes (a clade of tetrapod vertebrates comprising the reptiles, birds, and mammals who lay eggs on land or retain the fertilized egg within the mother), the alveolar lung of mammals may have been the earliest adaptation for land life, followed by its simplification in snakes and lizards [57]. There is no mechanistic basis for such speculation, as interesting as this idea may be. In fact, it stands in contrast to the presumed developmental pattern of the mammalian lung, believed to have begun as simple sacs that become progressively more structurally complex, consistent with the phylogeny of the lung evolving from the swim bladder of fish [58].

With this in mind, a cellular-molecular approach can be applied to the hypothetical role of physiologic stress in mammalian lung evolution to other amniotes with ‘simple’ lungs. The simple sac-like lungs of other amniotes is associated with a lack of an adrenaline response to corticoid-mediated stress due to the fundamental difference in the configuration of the adrenal glands in mammals versus other amniotes It is helpful here to note that the fish adrenal is composed of two separate organs for the elaboration of corticoids and catecholamines [59]. In mammals, the adrenal cortex lies on top of the medulla as a separate structure, and the corticoids secreted by the cortex pass down through the medulla, amplifying adrenaline production by stimulating Phenylethanolamine-N-Methyltransferase as the rate-limiting step in adrenaline synthesis [60]. In all of the other amniotes, such as birds, the chromaffin cells that synthesize catecholamines are interspersed within the cortical tissue and the relationship between stress and adrenaline production is not as well delineated [61].

Clearly, non-mammalian amniotes including birds evolved mechanisms to cope with the physiologic stresses of land adaptation. It would then seem no surprise that these extended into their adaptation for breathing as well. Unlike all mammals, birds have a ‘stiff’ lung composed of large air sacs [62]. The lungs are attached to the dorsal wall of the thorax during embryogenesis [63]. Furthermore, air entering the lung in those non-mammalian amniotes flows in only one direction, unlike the reciprocating nature of the mammalian lung [64]. Obviously, this indicates a fundamentally different way of adapting to air-breathing in birds. Alligators also exhibit the attachment of the lung to the chest wall during embryogenesis and in the adult in association with unidirectional airflow, in further support of the speculation that the fixing the lung to the chest wall during development is in service to the unidirectional flow of air [63,65]. This supposition is further supported by the fact that birds have blood glucose levels 10–15 times higher than mammals suggesting that instead of secreting fatty acids from fat stores in response to adrenaline for metabolic ‘fuel’ on an ‘as needed’ basis via the fight-or-flight mechanism used by mammals, birds are constantly in a ‘metabolically-on’ mode [66].

A noteworthy context of metabolic evolution is that both birds and humans are bipedal, which may have been a consequence of their both being endotherms. Being upright, both birds and humans have become much more metabolically efficient than cold-blooded organisms which require multiple isoforms of the same metabolic enzyme to survive at ambient temperatures, whereas endotherms usually have only one isoform [67]. Bipedalism may have resulted based on this metabolic advantage, freeing the forelegs to evolve into wings and arms through common genetic motifs [68].

## Physiological evolution is united by a cellular-molecular approach

The purpose of offering this type of detailed reconstruction of a pathway to endothermy which necessarily concentrates on lung evolution is that it illustrates the complex set of inter-linkages that must exist between any genetic shift, such as a significant duplication, and the series of consequential hormonal, cell-cell signaling and anatomic reverberations that ensue. A good deal of evolutionary research tends to concentrate on the adaptation of single traits, such as bird beaks or cichlid jaw shape. Yet, all evolutionary changes are inherently complex and coordinate. Since organisms are cellular, and organs are tissue ecologies, integrative action in physiology or phenotype is necessarily a cellular phenomena. As indicated in the case of endothermy, all adaptations in reaction to imposed environmental stresses require coordinated adjustments across an entire range of body systems. Therefore, evolutionary changes must advance through united cellular action at the level of local tissue ecologies despite our traditional concentration on macroorganic form. Phenotype can only eventuate from coordinate cellular action. In this way, a contemporary range of environmental experiences can be internalized, in part as epigenetic cues, to return to the unicellular zygote for its further adjudication in preparation for the next macroscopic re-elaboration [38,69–72].

In the past, this process would have been framed within the context of endless rounds of selection pressures by which an organism stumbles towards phenotypic adjustment. It is instead argued that a differing framework can be justified based within the now recognized capacity of cells as individual problem-solving agencies to act together as a consortia to mitigate stress [70–77]. The complex pathway to endothermy is just such an example, wherein, localized tissue ecologies, be they lung or adrenal, successfully reached new set-points, each meeting stress in its own way.

Certainly, any tissue ecology which is part of an entire macroorganism will have a set of functional relationships with that whole [78]. In specific circumstances, an organism-wide allostatic physiological adaptation might impose stresses on one or another specific sub-set tissue ecology within the whole. Naturally, any stress at the level of a whole organism must incur downstream effects. Under such a circumstance, specific tissue ecologies will adapt first at their own level as best as it can, seeking its own local ecological solution within the entirety. That tissue ecological unit will then act in its own behalf to attempt to re-achieve homeostatic balance. To do so, its primary stricture is that it must remain in concordance with those First Principles of Physiology which guide all cellular action [79]. Whatever changes that occur at that local ecological level must, in turn, have its own set of ramifications throughout the organism proper. Cellular-molecular adaptations that occur in one set of tissue ecologies, such as those detailed for endothermy at the level of the lung or adrenal, will have necessary ramifications on each of the other united tissue ecologies that comprise all macroorganisms. Consequently, each will need to compensate in its own manner adhering to its own requirement of the consistent maintenance of cellular homeostasis. It is analogous to the well-known phrase, ‘all politics is local’. Stress at the level of the whole requires adjustments at downstream tissue ecological levels. Adjustments at the level of a local tissue ecology then have its own reciprocating effects.

Each of the individual constituents of any localized tissue ecology is attempting to maintain its own homeostatic balance since this is the process that sustains them [25,70–72,75–77,80]. That balance point must lie within a bandwidth of acceptable flux deviations [72]. Together, individual cellular homeostatic boundaries enact the greater homeostatic set-point of the local tissue ecology of which they are constituents. Such homeostatic boundaries can be further considered as the most probable state that any individual cell or entire tissue ecology can be found [70–72]. It can be asserted that this preferential state is the one in which information transfer and energy efficiencies are both optimized for that particular set of collaborating cells. Since all macroorganisms are holobionts, this means that those participating cells in any tissue ecology, including all of our organs, include both our own individual eukaryotic cells and a participating. Therefore, those preferred homeostatic states at the level of the entire local tissue ecology can now be understood as the consensus parameters of both of those constituent fractions. It follows then that any ensuing metabolic output or explicit phenotypic expression emanating from that tissue ecology is itself a summation achieved through conjoint means [38,70,71,75,77].

In the past, cellular actions have been analogized to those of a biological automaton, akin to biological machines [81–84]. Contemporary research contradicts that viewpoint. Our cells and our companion microbiome exhibit a much wider range of capacities than had been previously considered in biological and evolutionary development. These faculties include the ability to access information, communicate abundantly, anticipate, predict and measure [70,71,74,85–98]. When combined into those tissue ecologies that comprise us, cells and microbes can collaborate, engage in mutualistic competitions, subspecialize, and trade resources [70,72,93,95,98–100]. In sum, all constituents of any macroorganic tissue ecology are effective individual and collective problem-solving agencies [70,72,73,77,101]. All our metabolic and physiological functions are the product of their interactions supported by a variety of sophisticated cell-cell signaling mechanisms [38,69]. Through these abilities and countervailing constraints, cells cooperate to sustain their collective state of homeostatic equipoise [34].

The concept of the senome has been recently introduced as the encompassing means by which cells acquire information and assess their environmental status [102]. The senome represents the summation of all the varied forms of sensing that individual cells employ that can be directed toward sustaining homeostatic equipoise. In essence, the senome can be considered a cell-wide sensory organ of assessment that links to those actions that permit continuous cellular-environmental complementarity by linking to the genome and epigenome to participate in cell-wide problem-solving. There is no doubt that such a connection is required since genetic or epigenetic responsiveness is always downstream to sensory inputs for any cell. Working in reciprocation, the senome, epigenome, and genome permit the entire spectrum of response from the reception of environmental information to the appropriate molecular deployments to meet threats and maintain homeostatic balance. It is now further argued that the senome concept of the cell can be productively applied to tissue ecologies. These ecological units are highly coordinated and co-dependent cellular collaboratives, so, it is reasonable to consider that any capacities that exist at the level of individual cells would be recruited to function at the multicellular ecological level to assist in tissue ecology-wide sensory responsiveness. It is proposed that tissue ecologies utilize a collective senome to assess their status within a holobiont, functioning at all stages of the life cycle from conception forward.

It is therefore being asserted that cells are not mere unknowing constituents of local tissue ecologies with only automatic reactions to genetic and hormonal cues. Instead, they assess, communicate and deploy information as purposeful and active participants in their own fate [70–72,77]. As Stuart Kauffman (2000) has aptly noted, all living things can act on their own behalf [103]. As living organisms, our cells can and do act in that same manner, whether that is during growth and development or over evolutionary space-time. This latter perspective has been codified as Cognition-based Evolution which emphasizes high levels of reciprocation between tissue ecologies and evolutionary development [70–72,77]. Thus, it is proposed that the evolution of the exteriorization of the testes should reflect the capacity of testicular cells to actively respond to endothermy and its imposed internal stresses, such as the requisite physiological adaptations that have been already enumerated, as collective adjustment through coordinate self-directed cell-cell signaling. The summation of the physiological, metabolic, and environmental milieu of each species should thereby directly link to optimization of testicular cellular dynamics given whole-organism constraints. One means of adjustment towards attaining that optimized status is location within the holobiont.

In such circumstances, testicular location is not mere selection default subject to eons of trial and error. Instead, it is a product of proactive cellular dynamics. The cellular direction is consensual homeostatic balance in the context of the unique spermatogenic environment that must be met to continue to render advantaged reproduction. Therefore, testicular location in varied species should be viewed as the active consequence of a unique tissue ecology with a self-aware constituency seeking its own best location to maintain its homeostatic requirements within a macroorganism. This coordinate cellular action is what is then subject to filtering selection. This becomes a function of cell-cell signaling, inter-relating endocrine pathways, energy efficiencies, rates of spontaneous genetic mutation in sperm, internal factors such as core body temperature and immunodynamics. When so blended, final mature location becomes an issue of the consensus of the cellular community ecology and its adaptive potential within that location.

## A case of common descent

There is no specific aspect of anatomy that provides a greater differentiation between males and females than the reproductive system. After a brief indeterminate period in humans lasting six weeks, the testes and ovaries originate from the same embryological tissues [24]. Beginning in week seven, the onset of the production of testosterone instigates the process of differentiation [7]. The ovary and testes both originate adjacent to the kidney, but the testes takes the long journey in most mammals. In humans, that transit involves at least two steps, a transabdominal phase and an inguinoscrotal passage [5,24]. Importantly, movement of the testes is relative. Its position shifts in a highly choreographed set of coordinate movements between the testes and other body tissues. Although it has been proposed that the abdominal cavity shifts and the testes is held in a generally rigid position by the gubernaculum, there is direct evidence that transabdominal testicular movement occurs as a combined push and pull interchange [24,104]. Crucially, the gubernaculum, which is more developed in the male fetus compared to the female provides a uniform trans-species pathway of testicular descent [105]. The female gubernaculum also assists ovarian descent and becomes the round ligament of the uterus as vestigial remnants.

The second phase of testicular descent, the inguinoscrotal passage, delivers the testes across the abdominal wall through the inguinal canal and then into the scrotal sac [7]. There is direct evidence that the gubernaculum is an active tissue participant during this phase. It swells in size and increases its diameter to enlarge the inguinal canal to permit the further passage of the testes [24]. That transit is both a push and a pull, partially relating to increasing intra-abdominal pressures. Once inside the scrotal sac, the gubernaculum becomes a connective tissue remnant and the inguinal canals close over it in humans [7].

The gubernaculum appears to be widely distributed across phylogenies. For example, nematodes and almost all mammals have one [106,107]. Yet, birds do not have a defined gubernacular structure. Their testes originate above their kidneys and stay there. Although bird testes share some commonalities with other mammals, their testes have scattered Leydig cells in the testicular interstitium rather than concentrated around the seminiferous tubules as is the case for mammals [108].

Given the above, it can be argued that the gubernaculum offers a degree of freedom within evolutionary development as a migratory route for the testes in mammals. The opportunity thereby arises to entertain the concept that the entire process is a complex balancing circumstance in which the constituents of the testicular cellular ecology determine its migratory path along a defined anatomical structure. Testicular location can then become a combination of ecological necessities, anatomic particulars, and complex physiological requirements. For example, testosterone signaling is imperative, but so too is insulin-3 which is responsible for testicular descent, masculinization and the outgrowth of the embryonic gubernaculum [109]. Non-endocrine molecules count, too. The temperature sensitive expression of molecular chaperone heat shock protein A2(HSPA2), a member of a heat shock protein family that has been evolutionarily conserved over metazoa, directly relates to levels of spermatogenesis [110].

**Figure 2.**
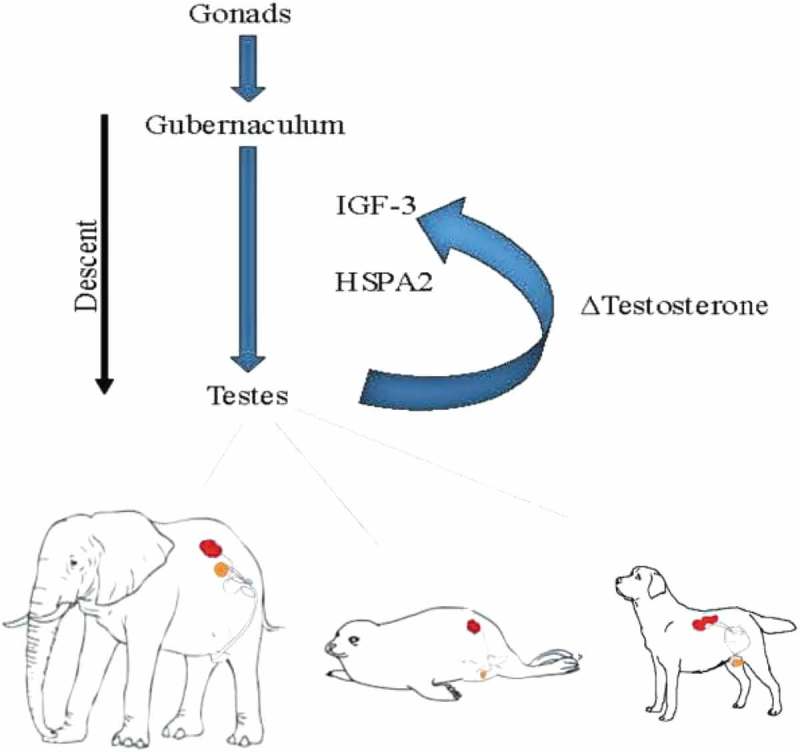
**Testicular descent**. The testes emerge from the common gonadal tissue and the gubernaculum determines the level to which they descend. The production of testosterone determines the length of the gubernaculum, mediated by Insulin-like Hormone (IGF)-3 and heat shock protein A (HSPA)2. Renal location in the diagram is indicated in red and final testicular location in orange.

Therefore, it can be argued that testicular location is the product of total complex molecular balance that is expressed across an entire tissue ecology that actively participates in its own location (Figure 2).

Organisms with a gubernaculum have the capacity to permit testes movement along an anatomical course to reach a consensual location for that cellular ecology and suits homeostatic requirements. Naturally, that location would be a reciprocal function of cell-cell communication with other cellular ecologies within the macroorganic entirety including the central genome. Although considering tissue ecologies in organisms as capable of proactive functionality is an unfamiliar frame of reference, all instances of testicular location can be rationalized by this means. As a particular example, in the elephant, there is no evidence of a gubernaculum, pampiniform plexus, processus vaginalis, or a scrotum [111]. Its mature testes can be found very near its peri-renal origin. Other animals have a gubernaculum, but show significant differences in mature location and in the timing of testicular movements. For example, testicular descent in rodents, rabbits and hares, and dogs takes place in the post-natal period [7]. In humans, pigs, cattle and sheep, testis position is settled before birth. It is asserted that the only means of rationalizing all these disparities of location and timing is to accept that testicular migration is based on cellular ecological cues that provide feedback to the genome. In effect, the processes of orchestrated cell-cell signaling that govern the descent of the testes in the biological development of any individual organism is a recapitulation of the process that has been applied to its location over evolutionary space-time. By this means, testicular location is not specifically selection driven. Instead, selection is the post-facto determinant that the migratory proclivities of the testicular ecology, based on its own cellular ecological measurements, is consonant with environmental constraints. In effect, selection assures that the cellular measurements of its homeostatic equipoise are correct and remain complementary to environmental necessities.

## Exteriorization based on evolutionary physiology linked to the origin of endothermy

By concentrating on the physiological steps in the evolution of endothermy as the continuous process of coordinate cell-cell signaling, a unifying perspective for testes location among animals can be offered. The key is to acknowledge that cells are not passive and can participate in their own fates. Certainly, too, they can coordinate to respond to internal physiological and external environmental stresses. Each cellular ecology does so in its own manner.

Instead of presuming that testicular location is the result of direct genetic instructions issued from a genomic bauplan that cellular ecologies passively obey, it is argued that cellular ecologies engage in an active process of reciprocation with the genome and epigenome to at least partially determine their own fates. At all times, this action is directed towards homeostatic equipoise at the local ecological level. The gubernaculum provides a scaffolding by which the testicular ecology and its supporting architecture can migrate towards their preferred environment in reciprocation with imposed physiological and environmental requirements of the whole organism in support of its total reproductive potential.

A unifying theory is thus proposed. Endothermy solved some problems with the external environment but imposed new internal stresses. The testicular cellular ecologies are able to assess a variety of environmental and hormonal cues and actively migrate towards a site of optimum balance over evolutionary space-time. This can be offered as the *‘Balanced Ecological Hypothesis*’. Testes originate in the abdomen in all animals and then descend to a variable extent to their final mature location. It is argued that this final location is a consensual cellular ecological balance point among the constituents of the testes tissue ecologies while necessarily remaining consonant with macroorganic reproductive requirements. Over evolutionary space-time, testes migration and its final location for each species has been the result of reciprocating cell-cell signaling with the general genomic bauplan, subject to filtering selection. It is proposed that the balanced homeostatic harmony of the cellular constituents of a specific tissue ecology can have a reciprocal influence on the genomic blueprint over evolutionary space-time. The genome is controlling but subject to reciprocal adjustment based on input from constituent cells. In the context of holobionts, it must even be considered that the testicular microbiome as constituents of the testes are participants in that signal driven process. Consequently, testicular location is not a simple result of selection pressures alone, but the product of the consensus of the entire cellular constituency of each testicular ecology, each according to species particulars.

In this manner, varied testicular locations rationalize. In each animal, its final location is that place within the entire macroorganism in which the homeostatic equipoise of the testes cellular ecology and its necessary appendages is best maintained. Therefore, its location is a function of all aspects of testes community ecology as it specifically inter-relates to macroorganic physiological realities and concomitant environmental influences. It is suggested that this is largely a function of information quality as a flow of variational free energy (a measure of the capacity to predict as the suppression of surprise) [112]. Energy efficiencies, hormonal interactions, and immunodynamics are other essential concerns.

It is therefore argued that the dramatic physiological changes in response to environmental pressures during the shift to endothermy for the water–land transition provided the relevant physiological backdrop to which the testicular ecological apparatus had to make accommodation. That adaptation could be expected to be twofold. Internal within the ecology to maintain cellular ecological stability and a permissive migration along the tract of the gubernaculum to a favored internal or external site of homeostatic equipoise specific to each species. Naturally, a significant aspect of part balance point is directed towards spermatogenesis. Barlow (2016) explains that sperm redundancy characterized by an enormous number of sperm per ejaculation is based on the need for sperm superfluidity to navigate the female reproductive tract. Importantly, total sperm cell count per ejaculate closely relates to testicular volume. In turn, testicular volume is best understood as itself an exaptation extending from the reproductive requirement for spermatic redundancy [113]. This reciprocating relationship between sperm number and testicular volume as phenotypic expression also supports the concept of reciprocating ecological balance which can then be applied to the further issue of testicular position.

The differences in testes location between birds and mammals become a dependent function of the disparate physiological background between birds and mammals. It is certainly not a specific matter of the dynamics of flight insofar as birds and bats both fly efficiently and bats have scrotal testicles and birds do not. What is clear, however, is that both birds and mammals evolved during the water–land transition, sharing unique traits of bipedalism and endothermy [21,114]. Bird physiology differs from mammals in three fundamental ways – their lungs are ‘stiff’, they are constitutively hyperglycemic, and the adrenal chromaffin tissue is dispersed throughout the cortical tissue of the adrenals [61,62,66]. These foundational physiologic traits, when placed in an ecological context, provide clues as to why the testes are internal in birds and external in most mammals.

It is known that the water–land transition was an adaptation to stress. Yet, it can also be assumed that just as some stresses were ameliorated during that passage, others were incurred. This rationalizes why it took many attempts to bridge that gap prior to its permanent success [37]. It has been advanced that the water–land transition was finally accomplished through known gene duplications for the Parathyroid Hormone-related Protein (PTHrP) Receptor, the Glucocorticoid Receptor and the βAdrenergic Receptor [27–29]. Amplification of the PTHrP pathway played a pivotal role in the evolution of the skeletal system, lung, and kidney [36,115,116]. The evolution of the Glucocorticoid Receptor was essential for both counterbalancing elevated blood pressure on land versus water [32] as well as in regulating the expression of the βAdrenergic Receptor [117]. And the duplication of the βAdrenergic Receptor was critically important for the evolution of pulmonary blood pressure separately from the systemic blood pressure [35].

In mammals, physiologic stress mediated by the HPA Axis causes increased catecholamine production from the adrenal medulla due to stimulation of Adrenocorticotrophic Hormone production by the anterior pituitary [118]. In turn, increased corticoid production by the adrenal cortex stimulates catecholamine production when passing through the adrenal medulla [50]. This increased catecholamine production by stimulating phenylethanolamine-n-methyltransferase (PNMT), the rate-limiting enzyme in catecholamine biosynthesis [60]. The resultant increase in catecholamine production due to physiologic stress during periods of intermittent hypoxia had a two-pronged effect. It primarily alleviated the constraint on gas exchange by the alveoli by stimulating surfactant production, allowing the alveoli to distend further and accommodate increased breathing. It was also permissive of circulating catecholamines stimulating lipolysis by fat cells, providing free fatty acids for metabolism, causing increase body temperature [53,119]. Ultimately this short-term adaptive mechanism was replaced constitutively by the production of oxytocin from the posterior pituitary [120].

It is proposed that one of the physiological forces contributing to the exteriorization of the testes in mammals was a secondary adaptation in response to the amplification of the βAdrenergic mechanism. This pathway stimulates the production of testosterone by Leydig Cells in the testes in the presence of pituitary luteinizing hormone [121,122]. Imbalanced production would lead to hypervirilized males. Exteriorized testes lowers testosterone production since there is decreased stimulation of testicular androgen production by catecholamines at 32°C than at 37 ^o^C, tempering testosterone production thus avoiding excessive virilization of male offspring [123]. Birds do not exhibit such stress-induced βAdrenergic stimulation of testicular androgen production because their adrenal medullary tissue is dispersed within the cortical tissue largely eliminating catecholamine stimulation by the adrenal [61].

This scenario is proposed as an example of the intricate interplay that reciprocates between tissue ecologies. Development of a male phenotype including virulization is driven by the overlapping influences by three hormones produced by the fetal testis in a biphasic pattern: anti-Müllerian hormone, insulin-like factor 3, and testosterone [7,124]. All of these morphogenic processes must interact with the overall genetic background of the organism across time and through combinations which are always unique within any species.

Testis development is unusual since it requires the overlap of several cell types including Sertoli, Leydig, and spermatogonial cells whose differentiation is co-dependent on Leydig cell signals and a range of transcription factors such as SRY and SOX9 [125]. Indeed, it even more complicated since testicular hormones are active in the nervous system, leading to neuropeptide secretion that has its own influence on the process of testicular descent [10]. For example, during inguinal passage, the genitofemoral nerve releases a neurotransmitter, calcitonin gene-related peptide, that serves as a chemotactic gradient to assist testicular migration [126].

That testes tissue has such a prominent migratory path could relate to its unique metabolic requirements. The only cells in the body that do not metabolize glucose are sperm cells [127]. They metabolize fructose as their prime energy source and fructose concentrations are considered a measure of seminal vesicle function and sperm motility [128,129].

It is thus argued that testicular position represents an instance of cell-cell signaling feedback with adjacent tissue ecologies, and ultimately the nuclear genome, to move the testes location to its point of substantial homeostatic equipoise in the temporal sequence that is consonant with the requisites of macroorganic reproduction. Its final location is that specific position that achieves a sufficiently balanced total testicular ecology including its supporting structures. The matrix of that decision process is a function of large numbers of hormones, transcription factors, cell-cell signaling, anatomic and mechanical necessities, and environmental factors, such as temperature. None of them have absolute primacy. The testicular ecology has a consensus balance point among its variety of cells, each with its own self-directed homeostatic needs. Together, they interact with the genome, metabolic substrates, and the macoorganic whole to achieve a settled position in an active manner that extends beyond simple selection biases across evolutionary space-time. Testes location rationalizes according to this overriding balancing mechanism. It can be in the scrotal sac in humans and pigs, in the abdomen near its site of origin in the elephants or birds, the subcutaneous tissues in pinnepids, or in the inguinal canal of the hedgehog [130].

## Effects of the failure of exteriorization of the testes on testicular ecologies

If the argument is made that testicular descent and its mature position reflect the necessity for a balanced cellular testicular ecology, then there should be some evidence that problems ensue if that ecological balance point is disturbed. The fate of maldescended testes can be offered in support of the Balanced Ecological Hypothesis. It is argued that the issues of impaired fertility and teratogenic potential that is seen in crytochordism are secondary to cellular ecological disruption, leading to deficiencies in tissue physiology and metabolism which can lead to a concomitant microbial dsybiosis.

Cryptochidism is a common congenital birth defect affecting between 2% and 5% of full-term male births [131]. The causes are not well established although there do appear to be links to disruption in Leydig-cell hormones including *Insl3* and testosterone [132]. Recent research has provided some indication of a genetic cause, including mutations in the genes for *Insl3* and its receptor [133]. A host of environmental factors have also been implicated [126]. For example, maternal exposure to estrogens downregulates *Insl3* expression in embryonic Leydig cells that can lead to cryptochidism [109].

It is well established that cryptochidism is associated with relative or complete impairment of fertility which can persist even after correction [131]. Even when surgically repaired, impaired fertility is evident in 33% of unilateral and 66% of bilateral undescended testicles [126]. Impaired fertility in undescended testicles can be correlated to the serious damage that these testicles demonstrate on histopathology [134]. Cryptorchid testes are much smaller than normal with atrophic tubules and sparse germinal epithelium and Leydig cells. In such a circumstance, there is straightforward and profound morphogenic tissue ecological disruption. In lesser degrees of impairment, research has demonstrated that more subtle disruptions of the testicular ecological niche. The testicular microenvironment including interstitial tissues play a critical role in spermatogenesis and spermatogonial stem cell function [135]. Spermatogenesis is the complex inter-relationship between the ability of tissue-specific stem cells to sustain regenerating tissue lineages in reciprocal dependence with supporting cell populations [136]. Therefore, there is a direct link between abnormal testis location and ecological tissue disruption with diminished spermatogenesis.

As opposed to infertility, the causes of the elevated incidence of testicular cancer is less well understood. It is known that there is a substantial increase in the incidence of testicular cancer in crytorchidism with a 5–10 times greater risk than in normal individuals [126]. There has been a general assumption that the development of testicular germ cell tumor (TGCT) reflects imbalances in endocrine control, particularly for androgen/estrogen levels [137]. Others have found a high rate of polymorphisms in receptor (ESR) genes and steroid hormone metabolism genes that might be associated with TGCT [138]. Interestingly, there is a strong association between the phenotype of TGCT stem cells and the fetal primordial germ cell [139]. This opens the possibility of examining TGCT pathogenesis as a combination of genetics, environmental exposures, and the testicular microenviroment. Rijaarsdam and Looijenga (2014) assert that the similarities between testicular germ cell cancer stem cell components and embryonic stem/germ cells imply a breakdown of cell-cycle control mechanisms and critical receptor tyrosine kinase signaling pathways that mediate oncogenesis within the testicular microenvironment [139]. Critical embryonic/germ cell micro-RNAs also appear to play a pivotal role in this process by regulating pluripotency and cell-cycle control. This inter-relationship is of particular importance. Primordial germ cells are the precursors to all gametes as the founder cells of the germline, and can be reprogrammed into both pluipotent stem cells or germ cell tumors, such as testicular teratomas [140].

It is now known that the testicle has its own microbiome and that it is a critical aspect of the testicular extracellular microenviroment [141]. Normozoospermic men demonstrate bacteria in their testes. Actinobacteria, Bacteroidetes, Firmicutes and Proteobacteria species are most common [141]. Others have found high levels of Prevotella and Lactobacillus [142]. Retrieved samples from men with idiopathic non-obstructive azoospermia and germ cell aplasia demonstrate microbial dybiosis patterns, with an increased number of Actinobacteria and Firmicutes in the former and lowered bacterial diversity and microbial numbers in the latter [141]. Therefore, it is now evident that testicular bacterial community types are directly associated with semen health and fertility [143]. It is noteworthy that there is a total absence of Clostridia in complete germ cell aplasia. Clostridia has been associated with human sperm motility. Furthermore, the four major phylogenies in normozoospermic testes are the same strains that predominate in the human gut. This is pertinent since it has been experimentally shown that the gut microbiome plays a crucial role in testes development, modulating blood-testis barrier permeability and endocrine function [144,145].

As at all sites, like the oral cavity, gut or skin, the intrinsic microbiome plays an integral part in the overall health of the total tissue ecology. Among many functions contributed by microbes in tissue, ecologies are immune responsiveness, levels of adaptive immunity and the protection of the ecology from pathogens [146,147]. Indeed, it is now understood that there is no metabolic pathway that does not rely to some extent on interactions between our intrinsic cells and our microbiome [148,149]. Although it had been previously thought that the great majority of cancers are due to genetic mutations, increasing numbers of cancers are now known to be caused by infectious disease. Microbes are now known to be a common cause of cancer including human papilloma virus (anogential cancers), Heliobacter pylori (gastric cancer), hepatitis B and C (hepatic cancers) and human T-cell lymphotrophic virus type 1 (adult T-cell leukemia/lymphoma) [150,151]. Importantly, this is not solely a matter of direct cancer induction at the level of the parenchyma of a target by an isolated microbial type. It can also be the result of a complicated set of direct and indirect microbial community interactions known to promote metaplasia in addition to metabolic changes. For example, Blaser (2008) suggests that indigenous colonic microbiota affects the metabolism of estrogens that enter the enterohepatic circulation [150]. Elevated circulating estrogen levels have been linked to cancers of the breast, ovary, endometrium and testicular malignancies. Others cancers are not directly tied to a specific infectious agent or a circulating metabolite, but may be related to microbial dysbiosis in specific tissue ecologies [151]. For example, Xuan et al., (2014) have demonstrated that human breast tissue has resident bacteria and human breast cancer is associated with a dysbiosis that influences the local immune microenvironment [152]. A similar type of microbial dysbiosis which has both direct and indirect effects has also been associated with prostate cancer. The local dybiosis affects hormone levels and induces local and systemic inflammatory states with subsequent immune dysfunction [153].

It is clear that there is no common agreement among researchers as to the cause of testicular cancers [154]. However, an accumulating weight of evidence suggests that the teratogenic effects of cryptochidism can be ascribed to ecological imbalance of innate testicular cells with their companion microbes as a generalized ecological dysbiosis which has reverberations at multiple system levels. Therefore, both infertility and increased teratogenic potential manifested in cryptochidism are reflections of testicular tissue ecological disruption, either primarily at the metabolic level or through disruption of its associated microbiome. In both circumstances, the essential ecological balance is upset. The level of intertwining intimacy between our microbiome and innate cells is only beginning to be understood, but it is becoming increasingly clear that they function together as an ecological community [155].

Therefore, it can be defended that both infertility and elevated rates of germ cell cancers in crytorchidism are both expressions of significant disruption of the testicular ecological balance. When the intricacies of the interactions of the united cellular types that constitute the testes are considered, any change in tissue ecological cell-cell signaling matters and there is no question that this is substantially influenced by testicular position. The further range of external influences can be myriad. These can include hormonal responsiveness, mechanical factors, or ambient temperatures. For example, varicoceles have been shown to be associated with elevated scrotal temperatures and a significant incidence of impaired spermatogenesis [156,157]. Clearly then, a large range of processes can disrupt the proper balance of any tissue ecology and it is their total harmony that is the common denominator of testicular vitality.

Certainly, any proposal that tissue ecologies engage in highly intricate coordinate signaling with other body tissues is not itself controversial. It is obvious that there is a highly choreographed interplay between developing fetal tissues and a genetic blueprint that is individualized for tissue types, each with its own temporal time-table. Rather than the prevailing assumption that any evolutionary changes in coordinate development are due to mutations and selection, it is defended that at least some tissue ecologies assist in their patterns of development within a holobionic entirety based on active and specific ecological feedback to the developmental bauplan. Using the testes as a model permits the testing and potential refutation of the hypothesis. However, if supported by further research, a substantial new evolutionary pathway will have been confirmed and can then be further assessed.

## Conclusion

A discrete pathway to the origin of endothermia has been outlined as illustrative of the types of intersectional adjustments that are required by organ tissue ecologies in response to macroorganic stresses which might then account for the variety of testicular locations exhibited across the animal kingdom. As that transitioning process unfolded, with some organ tissues attempting to ameliorate their own external environmental stresses, other organ tissues experienced countervailing internal constraints requiring their own intra-organismal accommodations. Although much of the external environment is experienced at the level of the whole organism, adjustments are necessarily enacted at the level of complex cellular ecologies in reciprocation with whole organism requisites. It is argued that the migration of the animal testes via the gubernaculum to a variety of internal locations and its exteriorization in most mammalian species is an example of this type of acclimation. The *Balanced Ecological Hypothesis* asserts that testicular location is the result of coordinated self-directed cell-cell signaling at the local tissue ecological level in support of local organ-specific homeostatic equipoise. In such circumstances, selection still operates, but is a post-facto filter of cellular ecological imperatives. In essence, capable cells measure and then act to sustain their homeostatic equilibrium. By this means, cellular ecologies actively participate in their own evolutionary space-time development to meet homeostatic requirements in a coordinate, non-random manner. In so doing, through their own self-directed participation in niche construction activities to sustain homeostatic equipoise, they exert a reciprocating influence on the general genomic developmental bauplan [70–72,77]. The focus on testicular migration and its ultimate mature location represents an example of how such an evolutionary process might work in response to larger organismal environmental pressures, such as the transition to endothermy.

Evidence that such a crucial local ecological balance point exists is provided by the high incidence of infertility and carcinoma in those testes that fail to reach an advantaged location. Once critical homeostatic interplay is lost, ecological dysbiosis supervenes within germ cells, supporting niche cells, the extracellular microenvironment, and its associated microbiome. That disruption is exhibited through observable morphological abnormalities, varying degrees of impaired spermatogenesis and elevated teratogenicity. Clearly, sustaining spermatogenesis and overall reproductive success is a complex organism-wide process. Through the consistent measurement of its own local ecological balance and by its vital organism-wide reciprocations, the testes are effective participants in that pivotal system and their own homeostatic equipoise.
